# Presumed Glenoid SLAP Tear in an Adolescent Football Player Belied the Presence of a Coracoid Growth Plate Fracture: A Diagnostic Conundrum

**DOI:** 10.1155/2022/1756611

**Published:** 2022-02-02

**Authors:** John G. Skedros, J. Hunter Marshall, John T. Cronin

**Affiliations:** Utah Orthopaedic Specialists, Salt Lake City, Utah, USA

## Abstract

We report the case of a 13-year-old male who sustained a right shoulder injury while playing quarterback in an American football game. A noncontrast MRI scan showed evidence of a possible grade 1 SLAP tear (SLAP = superior labral anterior-posterior), and surgery was recommended. However, at the preoperative visit, the surgeon was suspicious that the main source of pain had been missed. Bilateral shoulder radiographs taken for comparative analysis revealed a Salter-Harris type I coracoid growth-plate fracture. Therefore, the surgeon planned to manage the patient nonoperatively and postponed the proposed SLAP tear surgery. The patient was instructed to cease participation in athletic events and undergo physical therapy. Hoping to expedite his healing with platelet or stem cell injections, the parents sought an opinion from another physician who obtained a right shoulder MRI with intra-articular contrast. This confirmed that there was no SLAP tear. We concluded that the patient initially had glenohumeral and coracoclavicular ligament strains and a coracoid growth-plate fracture. The SLAP tear suggested by the initial MRI proved to be a red herring, belying an accurate diagnosis. The patient ultimately recovered fully with physical therapy and without surgery or other interventions.

## 1. Introduction

As participation in athletics increases among the pediatric population, reported cases of shoulder injuries are rising [1–3]. Therefore, the probability that a physician will see a patient with a relatively rare fracture or injury, such as a Salter-Harris type I fracture of the coracoid process, is also increasing. This case is presented to spotlight coracoid physeal injuries as a consideration when evaluating pediatric patients for shoulder pain.

Our patient is a 13-year-old male who was initially seen for a right shoulder injury after a fall. A SLAP tear was suggested on a noncontrast MRI scan. At the time of a preoperative evaluation for repair of this suspected lesion, the surgeon recognized the possibility that the patient might have a Salter-Harris type I fracture of the coracoid growth plate in addition to anterior glenohumeral capsular ligament and coracoclavicular ligament (CCL) strains. This fracture diagnosis was confirmed with bilateral axillary-lateral radiographs showing evidence of damage to the growth plate on the injured side and a nearly fused growth plate on the opposite side. He was managed conservatively.

## 2. Case Presentation

A healthy 13-year-old Caucasian male was referred to our clinic for persistent right shoulder pain after a fall in an American football game weeks earlier. At the time of the injury, he dove for a loose ball and fell on the lateral aspect of his right shoulder. He was unable to continue to play due to pain from the injury. There was no evidence of a shoulder dislocation. The patient was initially treated and monitored by his primary care physician, who felt after several weeks of persistent pain the patient should consult with an orthopedic surgeon.

The patient first visited our clinic 7.5 weeks after the injury. Physical examination revealed mild tenderness with palpation over and lateral to the coracoid process and with crossed body adduction and shoulder impingement maneuvers (Hawkin's and Neer). There was also a mild positive sulcus sign, and with the patient supine, there was mild anterior subluxation with passive abduction and external rotation of his right shoulder (i.e., positive apprehension test). Anterior-posterior, scapula-Y, and axillary-lateral radiographs of the right shoulder showed a patent growth plate that was not considered atypical at that time because of the patient's age. An MRI without contrast was ordered of the right shoulder for suspected injury to the joint capsule and/or labrum.

**Figure 1:**
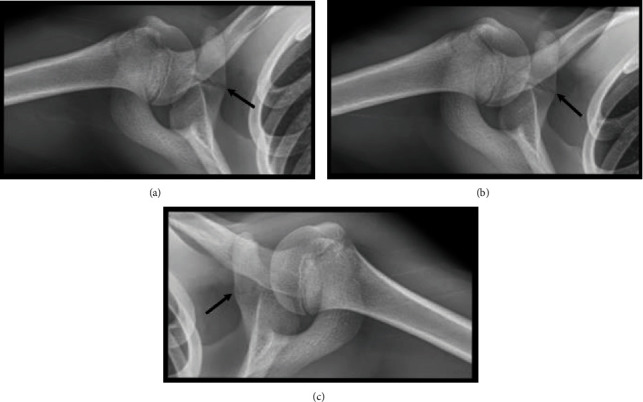
(a) Radiograph of injured shoulder taken at first clinical visit (12/27/2018) (7.5 weeks after the injury). (b) Radiograph of injured shoulder taken at the second clinical visit (1/31/2019) (approximately 11 weeks after the index injury). Note that the lucency extends across the entire coracoid. (c) Radiograph of contralateral (noninjured) shoulder taken at second clinical visit. Note that the lucency at the medial aspect of the coracoid is much less obvious when compared to the injured side.

**Figure 2:**
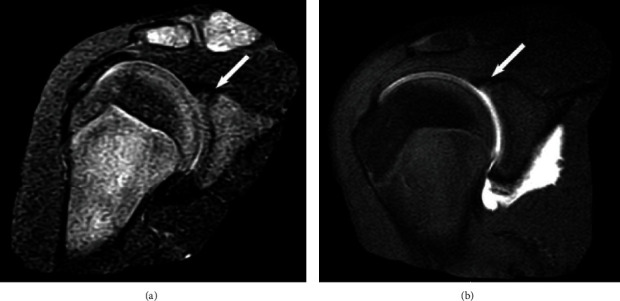
MR images of the patient's injured shoulder. Arrows point to the suspected SLAP tear in both images. (a) This image was taken approximately 11 weeks after the index injury. (b) This image is from the second MRI, which was approximately 27 weeks after the index injury. Because T2-weighted imaging was not performed during the second MRI scan, a direct comparison cannot be made to part (a). Also, the left (noninjured) shoulder was not evaluated with MRI.

The parents were anticipating that the next appointment would be to schedule shoulder surgery because they had seen the MRI report that stated that there was “irregular linear increased T2 signal within the superior labrum directed away from the glenoid” (Figure 1(a)). At that time, neither the radiologist nor orthopedic surgeon recognized the increased T2 signal in the bone near the coracoid growth plate might represent injury. Recognizing the rarity of SLAP tears in adolescents who did not have a shoulder dislocation, we tried to identify other potential sources of the patient's pain that had not been considered at the first consultation. Consequently, at his second visit to our clinic, radiographs were taken of each shoulder and showed a nearly fused main (basilar) growth plate of the left (non-injured) coracoid, while the right coracoid growth plate was still patent and had mild heterogeneity in contour suggestive of a Salter-Harris type I fracture (Figure 2). Notably, the epiphyseal plate of the coracoid process in normal healthy adolescents mirrors the contralateral side and generally closes symmetrically by 18 years of age [4, 5]. But bilateral asymmetry of closure of the main coracoid growth plate has been described in 3 of 131 (2.3%) modern skeletons in a sample from Portugal [5]. In this context, we concluded that different radiographic appearances of our patient's coracoids also supported the diagnosis of a nondisplaced growth plate fracture. His pain persisted primarily because of his continued attempts to participate in athletic activities in the setting of concomitant ligament injuries. He was enrolled in a physical therapy program, and a three-month follow-up appointment was scheduled.

During the interim between this last clinical visit and the next follow-up appointment, the patient again strained his right shoulder after falling on the playground at school. His parents sought a second opinion at an outside clinic that specialized in managing pain with injections, especially stem cells and platelet-rich plasma (PRP). They expressed concern to us that we were not treating their son as aggressively as they deemed necessary, and they also hoped that such injections might help enhance healing. At that other clinic, an MRI with intra-articular contrast of the right shoulder was ordered. This second shoulder MRI was 3.5 months after the first MRI. The region of the superior labrum that previously appeared to show a tear did not fill with gadolinium, indicating the abnormality was not a SLAP tear but rather a vascular or developmental variant (Figure 2(b)).

At the third visit to our clinic (four months after the first visit), another radiograph of the right shoulder was taken that showed that the injured coracoid growth plate was fusing. Physical examination showed mild anterior shoulder subluxation with mild subacromial pain. Continued physical therapy was recommended to address what we ultimately considered to be the most significant lingering problem (anterior subluxation from ligament stretch injury). The patient did not receive any supplemental injections. Three months later, he had no pain and was able to play quarterback in football without pain or any other limitations.

## 3. Discussion

This case highlights nuances that are important to consider when evaluating a suspected glenoid SLAP tear in an adolescent patient. The incidence and epidemiology of SLAP tears in adolescents have not been systematically studied and reported in a large number of patients. A few studies from pediatric hospitals have published retrospective case data regarding glenohumeral labral pathologies and generally report relatively low prevalence rates in this demographic group [6–8]. For example, Zbojniewicz et al. [8] reviewed 205 shoulder arthroscopy cases of patients aged 8-18 years and compared MRI findings to the intraoperative arthroscopic findings and found only nine (4.4%) cases with labral tears. The prevalence of glenoid labral pathology is most likely much lower [9] because this sample was biased towards shoulder pathologies. However, as participation in athletics increases among the pediatric population, reported cases of shoulder injuries are rising [1–3].

Diagnosing glenoid labral tears requires a combination of clinical evaluation and proper imaging techniques. Based on the mild sulcus sign and mild anterior subluxation, further evaluation of our patient's glenohumeral joint was warranted. However, as evidenced in our case, standard MR imaging may not provide the level of specificity that is required to correctly identify nonpathological labrums. MR arthrography (MRA) provides a more optimal visualization of intra-articular structures [10].

In patients aged <18 years, scapular fractures account for 0.33% of all fractures, with a peak incidence at the age of 14 years in males and 11 years in females [11, 12]. It is estimated that coracoid fractures comprise less than <10% of scapular fractures [11]. Ogawa et al. [13] segregated coracoid fractures into two main categories: type-1 is any fractures proximal to the coracoclavicular ligament (CCL) attachments, and type-2 is fractures distal to the CCL attachments. In pediatric patients, Ogawa type-1 fractures of the coracoid process predominate due to the relative difference in strength between the CCLs (relatively stronger) and physeal cartilage (relatively weaker). This explains why adolescents who play sports are at a higher risk of sustaining a coracoid physeal injury [11, 14]. Due to the very low prevalence and typically nondisplaced nature of these fractures, they often go undiagnosed upon initial evaluation. This is what happened in our patient.

Additionally, coracoid fractures are difficult to detect with typical radiographic views and require specific patient positioning for radiographic identification (e.g., angle-up view, anterior oblique view, or scapular-Y view) [11, 15–17]. When using these specialized views, radiography is usually sufficient for diagnosing acute coracoid physeal injuries [18].

Despite these potential diagnostic challenges, coracoid physeal injury should be considered when evaluating a pediatric patient for shoulder pain [19, 20]. As demonstrated by the clinical data reported by Mondori et al. [21], simultaneous fracture of the coracoid epiphysis and labral tear is not likely. Of the 37 patients that they describe with epiphyseal separation of the coracoid, none had injury to the glenohumeral labrum. Although these results are limited because only 10% of the cases reviewed underwent MRI examination, their findings are clear—the coexistence of a labrum tear and a coracoid growth plate is not likely in pediatric patients. They reported that the typical injury associated with a coracoid physeal injury is an acromioclavicular joint injury, which is what also occurred in our case. The grade 1 acromioclavicular strain that our patient had is also similar to the concomitant injury in the three additional case reports of patients 9, 13, and 14 years old [22–24]. These three additional cases also lacked evidence of significant labral injury. Generally, nondisplaced fractures of the coracoid are treated conservatively as are also most acromioclavicular injuries in adolescents [25–31]. Ogawa et al. [11] reported that 79% of pediatric patients with coracoid physeal injuries were successfully treated with conservative methods.

## 4. Conclusion

When evaluating a pediatric patient with posttraumatic shoulder pain, coracoid physeal injury should be considered in the differential diagnosis. This demographic is particularly susceptible to chondral shear injuries at the coracoid physis due to the relatively stronger CCLs pulling traction on the coracoid. The prevalence of SLAP tears in the pediatric population is exceedingly low, and the suspected presence of this injury in our patient's initial MRI scan belied an accurate diagnosis. He ultimately had a full recovery without surgery.

## Data Availability

Clinical data are available with appropriate request to the principal investigator.
